# On the Condition of Lunatics and Idiots in Ireland

**Published:** 1855-07-01

**Authors:** 


### Art. II.—ON THE CONDITION OF LUNATICS AND IDIOTS IN IRELAND.

A very curious, valuable, and highly interesting Special Report upon the "Number and Condition of Lunatics and Idiots in Ireland," as presented to Parliament in a General Report on the "Status of Disease" in Ireland, at the period of taking the last census for that country in March, 1851, is now before us.

Before proceeding to the more particular object of the Report, it will be instructive to take a glance at the state of Ireland, in reference to any provision for the accommodation of the insane at an early period in the history of that country. In 1701, a workhouse was erected and opened for the relief of the poor of the city of Dublin, under the control of the Corporation, which afterwards merged into a Foundling Hospital. Sir William Fownes, Bart., who had been, in 1708, Lord Mayor of Dublin—writing to Dean Swift in the year 1732—says, "When I was Lord Mayor, I saw some miserable lunatics exposed to the hazard of others as well as themselves. I had six strong cells made at the workhouse for the most outrageous, which were soon filled ; and by degrees, in a short time, these few drew upon us the solicitation of many, till by the time the old Corporation ceased (1728), we had in the house forty and upwards." And in addition he states, "there is no public place for their (lunatics) reception, nor private undertakers, as about London." In 1730, this building assumed the name of the Foundling Hospital and Workhouse of the City of Dublin, and is the first record of the erection of lunatic cells in Ireland. In 1711, by order of the Lord-Lieutenant, who at that time was the governor of Kilmainham Hospital (opened in 1684 for discharged invalid soldiers), and Sir Patrick Dun, physician of the same, cells were constructed adjoining the infirmary of that institution for the reception of such soldiers quartered in Ireland as might happen to be afflicted with mental derangement. These original cells, which were constructed in accordance with the cruel and unnecessary coercion then in use, for the custody, but not the treatment, of the insane, were thrown down in 1730, and others, more capacious and better suited to the wants of their unfortunate inmates, were erected. Two years afterwards, Sir William Fownes, in answer to a communication from Dean Swift, drew up a Proposal to the effect, "That an Hospital called Bedlam be built in the City of Dublin or Liberties, for the Reception of Lunatics from any part of the kingdom;" and in allusion thereto, wrote, "then I would have the cells at the Royal Hospital Infirmary—lately made for mad people, &c.—examined, how convenient, and how in all points they are adapted to the purpose, with the costs of those cells, which I take to be six or eight."

Next to the above, and the first of the existing institutions, both as to time and magnitude, is St. Patrick's Hospital, erected in accordance with the will of the celebrated Jonathan Swift, Dean of St. Patrick's,

"In which he bequeathed the sum of about 12,000*l.* to purchase lands, with the profits of which to erect and endow 'an hospital, large enough for the reception of as many idiots and lunatics as the annual income of the said lands, &c.," shall be sufficient to maintain. It is evident from Swift's writings, that he conceived the idea of giving

> . . . the little wealth he had,
> To build a house for fools and mad, so early as 1731, when he wrote the celebrated and characteristic verses on his own death. In the following year he communicated with Sir William Fownes upon the subject, who thereon addressed to him the proposal to which reference has been already made. In 1746, the year after his death, the executors of Swift's will were incorporated by charter into a body of governors, voluntary contributions to a large amount were collected, which, with parliamentary grants and the proceeds of the Dean's bequest, enabled the governors to erect the present hospital in the vicinity of Kilmainham, and to open it for the reception of fifty patients upon the 9th September, 1757."

Those buildings which were subsequently erected, up to the present time, are given in a very simple form in table No. 5, and page 60, of the Report, and accompanied with much interesting and useful information respecting the greatly increased accommodation for the Insane, both in public and private asylums.

We will next direct the attention of our readers to the very important points which are more prominently brought before our notice in this Report; and introductory thereto we cannot do better than quote the words of the Commissioners:—

"We now approach a subject on which we possess some means of comparison with previous times, as for a considerable period annual Reports have been presented to Parliament by the inspectors of lunatic asylums, and because it was one that was discussed at length in the Report upon the Census of 1841. Moreover, from the number of persons belonging to the class now under consideration, who are confined in properly regulated asylums and other public institutions, the information respecting lunacy and idiocy which has been afforded is much more exact than could possibly be obtained at any previous time.

"In order to arrive at the number of lunatics and idiots in Ireland at the time of taking the Census, a special return, 'Form I.,' Table I., requiring the names, *or initials of names*, the age, sex, marriage, rank, profession, and the occupation of each person when in health;—the state of education, peculiar character of insanity, duration and presumed cause of disease, as well as the native place, were procured for all the persons confined or under treatment in the different lunatic asylums in Ireland, as well as the lunatics and idiots in gaols and workhouses. It was also deemed advisable to make an effort to collect, as far as possible, similar information concerning those lunatics and idiots who were either at large or in the custody of their friends at the time of taking the Census. 'Form D.' was constructed for this purpose, and placed in the hands of the enumerators (chiefly constabulary and police), who were informed in the 'Instructions' that mendicants and vagrants of these classes should be described from the best local information they could acquire; and we added, 'where lunatics or idiots are in the custody of their friends, the inquiry necessary to fill this form should be made with *the greatest delicacy.*' These Returns were subsequently verified by the enumerators.

"Although this is the first time that a similar inquiry has been instituted in this country, we are happy to say that no difficulties were thrown in the way of the enumerators, and that returns were made upon these forms of 1073 lunatics and 3562 idiots. These have been classified by counties and cities as lunatics and idiots 'at large' in Table I., pages 50, 51. In some cases the numbers specified in that and other tables given in this Report differ somewhat from those afforded by the inspectors of lunatic asylums in Ireland in their Fifth Report, published in May, 1851. In the Census previous to the present, no attempt was made to enumerate the lunatics and idiots at large or in the custody of their friends, but in the Report already referred to a return is given of the unaccommodated idiotic and insane for the different unions in Ireland, by which it would appear that 3674 idiots and 931 lunatics, besides 4380 epileptic imbeciles, making in all 8985 of this class were then at large or in the custody of their friends. That return was made through the Poor Law Commissioners, by the relieving officers of each electoral division, but the estimate entirely rests on the authority of these officers, who merely stated the numbers they believed existed in their districts without specifying the names, ages, sexes, places of abode, or other circumstance of such persons—all of great importance, if not indispensable, in arriving at the numbers and condition of any class of the community."

In the Tables which accompany this Report, is comprised information of an extremely valuable description, both for present use and for future comparison, and of the general objects of which tables we will give a short explanation.

Table 1 contains the number and distribution of the insane and idiotic in Ireland, arranged according to the provinces—viz., Leinster, Munster, Ulster, and Connaught, and each subdivided into its respective counties.

Table 2 presents at one view—

1st. The Occupations of Lunatics;

2nd. The presumed Causes of Insanity, which are placed under these four general heads—viz., Moral Causes—Physical Causes—Hereditary Taint—Causes which are not specified.

Table 3 gives a classification of the Insane and Idiotic in reference to the particular form of the Disease, and are arranged under these heads:—Mania—Suicidal Mania—Monomania—Dementia—Dementia with Epilepsy—Dementia with Paralysis—Idiocy—Unspecified Diseases.

And in further illustration of the preceding Tables, the Commissioners have added a 4th Table, containing 1st, the Ages—2nd, Married—3rd, Unmarried—4th, Educated—5th, Uneducated.

It appears by the tabular statement that there were "confined in the Irish asylums, prisons, and workhouses," 9980 Lunatics and Idiots, which are thus divided:—

| | | |
|---|---|---|
| Lunatics—Males . | 2503 | Total 5074 |
| " Females . | 2571 | |
| Idiots—Males . . | 2666 | Total 4906 |
| " Females . | 2240 | |

and in addition thereto, under the head of "At Large, or in the Custody of their Friends,"—are 1073 Lunatics, composed of 554 males and 519 females, thus making the whole number of insane, 6147.

To give our readers some idea of the very great pains taken in this Report, we will offer in some detail a description of a portion of Table

No. 2, in respect of the occupations of lunatics, as it comprises a very elaborate classification, under ten principal classes, and which are again very numerously subdivided.

The principal classes are thus described:—1st, Professional; 2nd, Professional with Mercantile Pursuits; 3rd, Literary and Educational; 4th, Shopkeepers and Traders; 5th, First-class Trades; 6th, Second-class Trades; 7th, Agricultural; 8th, Occupations producing Exposure; 9th, Special Female Occupations; 10th, Unclassified.

And also as regards the presumed causes of insanity, under its first specified division already mentioned—viz., "Moral or Mental Causes." This is formed into the following fourteenth subdivision—viz., Grief—Reverse of Fortune—Love and Jealousy—Terror—Religious Excitement—Study—Anger—Ill-treatment—Anxiety—Pride and Ambition—Political Excitement—Music—Joy—Remorse.

And to enable our readers the better to appreciate the information contained in this very curious and valuable Table to which we have just been alluding—we will place in a short tabular form an analysis of the results obtained from the "Presumed Causes of Insanity."

| | Males. | Females. | Total. |
|---|---|---|---|
| 1st. Moral or Mental Causes . . | 370 | 477 | 847 |
| 2nd. Physical Causes . . . . | 560 | 394 | 955 |
| 3rd. Hereditary Taint . . . . | 169 | 194 | 363 |
| 4th. Unspecified Causes . . . | 4070 | 3746 | 7816 |
| Total of Insane . . . | | | 9980 |

Adopting the same Tabular Form, we will also show at one view the number of insane belonging to each division as above given, and compared with the ten classes of occupations already spoken of.

| OCCUPATIONS. | CAUSES. | | | |
|---|---|---|---|---|
| | MORAL. | PHYSICAL. | HERE-DITARY. | UNSPECI-FIED. |
| | Male & Fem. | Male & Fem. | Male & Fem. | Male & Fem. |
| 1st Class, Professional ...... | 40 | 54 | 31 | 279 |
| 2nd Class, Ditto, with Mercantile Pursuits ......... | 9 | 4 | ... | 9 |
| 3rd Class, Literary and Educational ........ ... .. .. | 40 | 11 | 5 | 44 |
| 4th Class, Shopkeepers and Traders ................. | 25 | 13 | 6 | 66 |
| 5th Class, First-class Trades. | 32 | 37 | 14 | 78 |
| 6th Class, Second-classTrades | 78 | 76 | 16 | 251 |
| 7th Class, Agricultural ...... | 155 | 162 | 68 | 1213 |
| 8th Class, Occupations producing Exposure ...... | 15 | 22 | ... | 45 |
| 9th Class, Special Female Occupations ............... | 44 | 20 | 14 | 142 |
| 10th Class, Unclassified ...... | 409 | 555 | 209 | 5689 |

To draw attention still more strongly to the general results from these very valuable Tables, we cannot do so more effectually than by giving at length the words of the Report.

"In enumerating the occupations of the deaf and dumb, we only learn what amount of industrial education that afflicted class are susceptible of. In instituting a similar investigation for the blind, some information may be gleaned with respect to the influence of occupation in the production of disease, but in studying the circumstances which tend to the propagation of lunacy or idiocy, it becomes a matter of great importance to observe the influence which the various occupations and modes of life have in inducing these affections. We have therefore endeavoured to arrive at as much accuracy as was possible with respect to the previous modes of life among lunatics and non-congenital idiots. The results have been classified and arranged in Table II., pages 54 to 57, in which have also been grouped the various causes, both moral and physical, which, according to the returns received, were believed to have been the immediate or exciting cause of disease in this class. In that Table are included the entire number of lunatics and idiots enumerated on Table I., but of these, 2164 only have had the causes of mental alienation assigned, the remainder being either unspecified, or concerning whose previous condition no accurate information could be procured—many of them being wanderers and mendicants. In arranging a classification of such occupations as might, in some measure, have conduced to the propagation of mental disease, difficulties arise which do not occur in drawing up a classification of the general occupations of the community where several modes of life are grouped together, not as they affect the physical or moral condition of the individual, but as they minister to the general wants of the people.

"In the following Table, ten divisions have been made. The first consists of the professional class, of whom 404 were affected with insanity, a large amount, considering the proportion which this class bears to the great bulk of the people, and exceeding by a considerable number all the other specified classes, with the exception of the agricultural. This preponderance of mental disease among the professional and upper classes, shows how much more education and habits of thought tend to produce aberration of intellect than ordinary manual labour. In this class, disease was attributed to moral causes in 40 instances, and in 54 to physical. Among the former—grief, study, and reverse of fortune were the causes to which it was chiefly assigned; and in the latter intemperance prevailed to a great extent. In 31 instances the disease was attributed to hereditary taint, and in 279 cases no cause was assigned.

"Among the professions we find the following numbers affected with mental disease:—clergy, 36; officers, including those of the army, navy, and police, 34; lawyers and attorneys, 28; and medical men, 13. Of the 404 persons in this first class, 151 were females, of whom 148 belonged to what is termed the middle and upper ranks of society, specified in the Table as 'gentleman or lady.'

"In the second class are included those engaged with professional and mercantile pursuits; consisting of occupations which engage a certain amount of mental labour, together with the employment of capital, influenced by the ordinary fluctuations of trade and merchandise. This division numbers but 22, being the smallest of the entire.

"The third class we have termed the literary and educational, consisting of those engaged in mental occupations, either of acquiring or imparting knowledge. It includes students and teachers of different descriptions, and numbers 100—of whom 65 were males and 35 females. The predominating morbid influence among this class is believed to have been study, of which 16 cases are recorded among the moral or mental causes.

"The fourth class, denominated shopkeepers and traders, embraces all those persons engaged upon a minor scale in trade or merchandise, together with petty dealers of different descriptions. It numbers 110—of whom 86 were males and 24 females; of the specified occupations, 71 were shopkeepers, and 27 provision dealers and huxters. Where the causes have been assigned, reverse of fortune among the moral, and intemperance among the physical, predominated.

"In the fifth class are included first class trades; it numbers 161 persons, 158 males and 3 females. The most numerous occupations were clerks, 73; and cabinet-makers and carpenters, 61; grief among the mental, and intemperance in the physical causes prevailed.

"In the sixth class are enumerated second class trades, among whom the most numerous were weavers, 96; boot and shoe makers, 76; tailors, 48; and smiths, 29. Masons and bricklayers, painters, butchers, and flax-dressers also afforded many instances of insanity. The entire number in this division is 421 persons—391 males and 30 females, the latter consisting of confectioners, and persons employed in weaving and the manufacture of flax and wool. In 78 instances moral causes have been assigned; and of these, grief, reverse of fortune, love and jealousy, terror, and religious excitement predominated; and in 76 cases the insanity was attributed to physical causes, among which intemperance, fever, and injuries of the head were the most numerous.

"The seventh class consists of all those persons more or less engaged in employments contingent upon agriculture, general and out-door labour, and other pastoral and rural pursuits. It is, as might be expected from the occupations of the great mass of the inhabitants of this country, the largest division, numbering in it 1598 individuals—1496 males and 102 females; the females consisting of landholders, dairymaids, and farm servants, the latter being thus distinguished from the female household servants enumerated in the tenth class, and who number 635. Of those specified, 155 were attributed to mental causes, such as grief, reverse of fortune, terror, love and jealousy, anger or excess of passion, and religious excitement;—162 to physical influences—and of these, intemperance, congenital malformation, epilepsy, fever, the effects of climate, injuries of the head, and the deleterious effects of mercury, have been recorded as the most frequent; and 68 were assigned to hereditary predisposition.

"In the eighth class are included 82 persons, whose occupations necessarily induce considerable exposure, such as seafaring men, coach and car drivers, constabulary, &c. In this division, grief among the moral causes, and intemperance among the physical, are said to have produced mental disease in the greatest number of instances.

"The ninth class consists of special female occupations alone, and numbers 220; among whom 44 are said to have become deranged from moral causes, as grief, reverse of fortune, terror, love and jealousy, and ill-treatment. The physical causes assigned amount to only 20, of which intemperance, epilepsy, and fever were the chief.

"The tenth class includes such occupations as could not well be classed under any of the foregoing heads, such as billiard markers, lodging-house keepers, mendicants, &c., and also the unspecified. It numbers 6862 persons—2616 males and 4246 females—but of these 5675 were unspecified, chiefly idiots. Among the specified occupations or employments, that of servants amounted to 698—mendicants numbered 347, soldiers 93, and factory workers 41. Of this class 409 became lunatic or idiotic from mental causes, of which grief, reverse of fortune, love and jealousy, terror, religious excitement, anger, and ill-treatment were the most numerous. In 555 cases the assigned physical causes were chiefly congenital malformation of head, intemperance, epilepsy, fever, puerperal affections, the effects of climate, paralysis, disease of brain, and uterine derangements. In 209 cases the disease is said to have been hereditary.

"From an examination of the totals of Table II., we find that a return has been made chiefly upon medical authority, or by the governors of the various asylums, of 2164 cases in which the cause of disease has been investigated, and an opinion offered thereon. The total cases assigned to physical causes amount to 954, the sexes being as 100 males to 70·36 females;—those to moral causes number 847, in which class the reverse obtains as respects the sexes, for there we find the females predominating as 128·92 to 100 males. Those instances believed to result from hereditary predisposition are 363, the females being in the proportion of 114·79 to 100 males.

"In both sections the causes have been arranged in numerical order; thus among the moral or mental we find grief predominates, being about one-third of the whole, and the sexes being 195·7 females to 100 males; reverse of fortune, 170; the sexes being 100 males to 86·81 females; love and jealousy, 106, the sexes being 67 females to 39 males; terror, 101, or 62 females and 39 males; religious excitement, 55, or 25 males and 30 females; study, 37, or 35 males and only 2 females; anger or excessive passion, 32, the sexes being equal; ill-treatment, 28, or 22 females to 6 males; anxiety, 24, or 14 males to 10 females; pride and ambition, only 9, or 5 females to 4 males; political excitement, 6, or 5 males and 1 female; music, 2 males; joy, 1 male; and remorse, 1 female.

"Among the physical causes, we find 351 cases attributed to congenital disease, specified as malformation of head, and composed chiefly of idiots, the great majority of whom might with justice be classed with those set down under the head of hereditary taint; the sexes are in the proportion of 100 males to 94 females. In 216 instances intemperance is said to have been the cause of insanity, the male sex predominating so largely as to present the proportion of 100 to 21·35 females. Epilepsy numbers 100, the sexes being 56 females to 44 males, but this disease is, in many instances, only a symptom, and both it, disease of the brain, and paralysis, might all be classed together, when they would amount to 148:—the numbers attributed to fever are 81, or 42 males and 39 females; to injuries of head 39, or 33 males and 6 females; puerperal mania 36; the effects of climate, including sun-strokes, 33, chiefly males; disease of the brain (not produced by accident, including paralysis, which is but a symptom of the cerebral affection), 48, or 29 males and 19 females; the effects of mercury, 19, or 16 males, and 3 females; uterine derangement, 11; venereal excess, 7; dyspepsia, 7; cases attributed to rape and seduction, 4; and to violent hysteria, 2.

"Cases attributed to hereditary taint or family predisposition have been placed in a separate column, owing to the difficulty of determining how far the inherited peculiarity partook of the moral nature, or of the physical character transmitted from one generation to another. They number 363, or 194 females and 169 males. In 7816 instances the causes were not specified in the returns."

Upon the subject of the Classification of Insane and Idiotic according to the form of the Disease, the Report gives an analysis.

"In Table III. the form of disease, with the supposed cause, are grouped together, and the latter arranged according to the frequency of each description of excitement or physical disease. Collected from such a variety of sources, and the returns filled by persons varying much in intelligence and knowledge, these causes and descriptions of disease can be but approximations to the truth, still they are of value, not merely of themselves, but as a means of comparison for future investigations. The idiotic amount to 4906, the great majority of whom having been so born, no information could consequently be obtained with respect to the causes which induced disease. In 382 idiots who became so after birth, the causes are specified. Among the insane, mania was the form of disease manifested in about four-fifths of the whole; of these 669 instances were induced by moral, and 400 by physical causes, while 222 were attributed to hereditary taint. In 44 cases the mania was of a suicidal character, grief and reverse of fortune being the chief causes which conduced to this phase of disease. Out of 417 persons affected with dementia, in 73 cases the disease was attributed to moral, and in 69 to physical causes; while in 32 it was traced to hereditary predisposition—the remainder of each of these classes being unspecified. In 277 cases of dementia, 169 males and 108 females were also affected with epilepsy; and 15 other cases, 10 males and 5 females, of the same form of mental aberration, were likewise paralytic."

Another point of very considerable importance has also attracted the attention of the Commissioners, and they have placed in Table No. IV.

—under the heads already enumerated, describing the diseases in general terms of the insane—the ages of them, whether married or not, and also whether educated or not, and thereupon observe—

"Table IV. exhibits the ages of all the persons specified in the previous Tables, together with their state of marriage and education. From this we learn that of the entire 9980 persons, 1721 were married—647 males and 1074 females. In some few instances the particulars as to the state of marriage and education could not be discovered.

"In 3705 instances 3143 insane, 562 idiotic, and 69 unspecified—the sexes being 1996 males and 1709 females—the persons so affected were educated according to their respective ranks in life; and from this division of the Table we learn that, exclusive of the idiotic, the proportions were 100 educated to 61 uneducated, which, compared with the proportion which the educated bear to the ignorant throughout the general mass of the community, confirms the opinion with respect to the more educated class being more liable to mental affections than the unenlightened.

"It is only by a close examination of this and the foregoing Tables that the result of this minute inquiry can be seen or appreciated. The terms used for expressing the different forms of disease in these Tables are those employed in the returns afforded by the several public institutions, and as such, whatever may be their value, we are compelled to adopt them. We feel, however, that it is sufficient in this Report to afford, in the most comprehensive arrangements, the information returned in answer to our inquiries—leaving it for special writers upon this class of disease to avail themselves of the facts stated in these statistical returns as they think fit. We would, however, suggest to those who have the care of the insane, a more careful inquiry into the cause and form of disease under which patients labour, by which means, upon the next inquiry of this nature being undertaken, so large a proportion of the unspecified shall not appear. It is to be hoped that many of those returned on 'Form D.' at their own homes, in the custody of their friends, or who were wandering through the country, shall, before an inquiry similar to the present is again undertaken, be provided for in establishments suited to their wants."

In addition to this Return, the Commissioners have extracted the following information, contained in a Return made by Wm. B. Drury, Esq., Clerk of the Custodies in the matter of Idiots and Lunatics, in the Irish Court of Chancery.

"That 108 persons of weak or unsound mind—viz., 76 males, and 32 females, were under the control of the Irish Court of Chancery, in asylums, or under the care of friends or guardians, in March, 1851, and of whom 13 were resident in England."

The Commissioners give, as a general conclusion from the evidence contained in the Report, their opinion as follows:—

"Compared with the total numbers of the insane, mania prevailed chiefly in the counties of Monaghan, Tipperary, Longford, King's,

Donegal, and Londonderry; and the suicidal propensity seemed to be developed most in the counties of Carlow, Wicklow, Kildare, Kerry, Armagh, and the city of Limerick. Monomania was most prevalent in the cities of Limerick, Kilkenny, and Dublin, the town of Drogheda, and the counties of Kilkenny and Kildare; whereas, dementia prevailed most in the cities and counties of Waterford and Dublin, the city of Kilkenny, and the counties of Meath, Galway, Sligo, Roscommon and Limerick. In making these calculations, the various persons confined in the different asylums have been included according to their county or native place."

We conclude our analysis of this important Report with the general summary of the Commissioners.

"The total number of patients confined in all the lunatic asylums of Ireland in March, 1851, numbered 3436; or 1739 males and 1697 females, being 227 more than were returned in similar institutions in 1841. There were 173 lunatics, idiots, and epileptics in the different gaols and prisons, on the 6th June, 1841, and upon the 30th March, 1851, 286 of the same class were confined in similar establishments. 494 lunatics, and 1129 idiots were located in the different workhouses on the 30th of March, 1851; but no comparison can be made as regards the numbers of a similar class in 1841, as at the time of taking the Census of that period only two or three workhouses were in operation.

"From the foregoing enumeration, we learn that 5345 of the lunatic and idiotic class were under restraint in Ireland at the time of taking the Census, and of these 3436 were in establishments specially erected and intended for their treatment as well as their custody and support."

